# Socioeconomic patterns of overweight, obesity but not thinness persist from childhood to adolescence in a 6 -year longitudinal cohort of Australian schoolchildren from 2007 to 2012

**DOI:** 10.1186/1471-2458-14-222

**Published:** 2014-03-04

**Authors:** Jennifer A O’Dea, Hueiwen Chiang, Louisa R Peralta

**Affiliations:** 1 Faculty of Education & Social Work, University of Sydney, Building A35, Sydney, NSW 2006, Australia

**Keywords:** Longitudinal, Socio-economic status, Weight, Overweight, Obesity, Youth, Children, Adolescents, Social disadvantage, Social determinants

## Abstract

**Background:**

The prevalence of childhood overweight and obesity increased during the 1980s to the late 1990s. The prevalence of obesity is higher in socially and economically disadvantaged communities in most Westernised countries. The purpose of this study was to examine how the socioeconomic gradient in weight status, namely thinness, overweight and obesity, changes over time in a longitudinal cohort of Australian schoolchildren, from 2007–2012.

**Methods:**

939 Australian children in school grades 2–6 from 10 primary schools initially participated in the study in 2007. Height and weight were directly measured by research assistants each year. Obesity/overweight and thinness were defined by using the International Obesity Task Force BMI cut-offs. Chi-square analyses were used to test associations between categorical variables and linear mixed models were used to estimate whether the differences in SES groups were statistically significant over time.

**Results:**

Results found both males and females in the low SES group were more likely to be obese (6-7%) than middle (4-5%) and high (2-3%) SES groups and this pattern tended to be similar over the 6 year study period. There appeared to be no particular SES pattern for thinness with all SES groups having 4-5% of participants who were thin. The gender and SES patterns were also similar over 6 years for BMI with low and middle SES participants having significantly greater BMI than their high SES peers.

**Conclusions:**

Patterns of obesity and overweight in children from socially and economically disadvantaged communities in regional NSW are identifiable from a young age and the socioeconomic pattern persists into adolescence. Obesity prevention and intervention programs should be designed, implemented and evaluated with the social determinants of health in mind and in collaboration with community members. Community programs should continue to be based on positive rather than negative messages in order to avoid unintended stigma and other potentially harmful outcomes.

## Background

The prevalence of childhood overweight and obesity increased in Westernized countries during the 1980s to the late 1990s [1-5]. Potential links to childhood overweight and obesity include elevated risk of health concerns, including cardiovascular disease, Type II diabetes, some pulmonary and musculoskeletal complications [6-9] and a potentially increased middle-aged morbidity and mortality [10-12].

The recent prevalence of overweight and obesity in developed and developing countries suggests a continuation of this trend [13] but recent studies have reported a plateau in childhood and adolescent obesity prevalence in many developed countries in Europe [14-17]. Two recent reviews also found no significant increase in the prevalence of childhood overweight and obesity during the past two decades in nine countries including the USA, Australia, Netherlands, China, England, France, New Zealand, Sweden and Switzerland [18,19]. In particular, Australian data suggests a significant plateau in the prevalence of both overweight and obesity since the late 1990s [20-22].

While rates of childhood overweight and obesity appear to be stabilizing at present, in many countries they are still considered to be high for specific sub-groups within those populations. Global surveillance studies indicate there are age, sex, ethnic and socioeconomic disparities in the prevalence of obesity among youth, and that these disparities are similar across Westernised countries [14,16,17,23-27]. For example, a persistent relationship between low SES and increased overweight and obesity prevalence has been reported in several studies focusing on Australian youth [20,28,29]. One early cross-sectional national study of 5,000 children and adolescents in 2000 found that those from low SES schools were significantly more likely than their middle or higher SES peers to be obese (9.0 vs. 5.8%) or overweight (19.0 vs. 16.8%) and low SES predicted the risk of high body mass index (BMI) [30]. Worldwide, more recent studies have identified low SES as an important determining factor in childhood obesity prevalence and confirm this trend in several developed countries such as England [17], Denmark [15], Germany [24], Japan [31], the Netherlands [16] and Switzerland [14].

The main objective of this six year longitudinal study was to observe the long term patterns of thinness, obesity and overweight to examine how the socioeconomic gradient in weight status changes over time.

## Methods

### Participants

Participants were 939 Australian students from 10 primary schools in regional New South Wales school grades 2–6 who were enrolled in the National Youth Cultures of Eating Study (O’Dea 2008) study in 2007. A description of participants’ demographic and baseline (Year 1 of the study) characteristics from 2007 is given in Table 1. Initial parental and child consent and complete data sets for measured height and weight were provided from a total of 939/1010 participants who were enrolled in year 1 (2007) (479 male, 460 female) which reflected an initial 93% response rate. Retention of the initial cohort at each annual follow up was 87% in Year 2 (2008); 81% in Year 3 (2009); 79% in Year 4 (2010); 82% in Year 5 (2011) and 74% in Year 6 (2012). The participant’s ethnicity was self reported and included categories of Caucasian/Northern European (59.6%), Aboriginal/Torres Strait Islander (3.4%), Southern European (28.6%), Asian (Chinese/South East Asian, 3.5%), Middle Eastern (3.1%), Pacific Islander (0.7%), Indian or Sri Lankan (0.9%) and African (0.2%).

**Table 1:** Demographic, anthropometric and socio-economic characteristics of a cohort of Australian schoolchildren at baseline in 2007

	**Male (N = 479)**	**Female (N = 460)**
Age (years) (mean (SD)	10.1 (1.4)	10.0 (1.4)
School grade % (N)		
Grade 2 - 8–9 years	19.2 (92)	22.0 (101)
Grade 3 - 9–10 years	20.0 (96)	19.1 ( 88)
Grade 4 - 10–11 years	24.2 (116)	21.3 ( 98)
Grade 5 - 11–12 years	20.0 (96)	19.8 ( 91)
Grade 6 - 12–13 years	16.5 (79 )	17.8 (82)
Socioeconomic status SES		
Low	33.4 (160 )	38.7 (178)
Middle	33.6 (161)	31.1 (143 )
High	33.0 (158 )	30.2 (139 )
Father’s education		
Schooling to year 10 or less	25.3 (109 )	23.9 (96)
Schooling to year 12	11.8 (51)	11.7 (47)
Technical College	36.2 (156 )	35.8 (144 )
University Degree	26.7 (115)	28.6 (115)
Ethnicity/Cultural background		
Caucasian/ European	59.7 (286)	59.6 (274 )
Aboriginal	2.9 (14 )	3.7 (17)
Southern European	30.9 (148 )	26.3 (121)
Chinese/South East Asian	2.9 (14 )	4.1 (19)
Middle Eastern	2.1 (10)	4.1 (19)
Pacific Islander	0.4 (2)	1.1 (5)
Indian/Sri Lankan	0.8 (4)	0.9 (4)
African	0.2 (1)	0.2 (1)
(Mean (SD)		
Height (cm)	140.7 (0.09)	140.4 (0.10)
Weight (kg)	36.6 (8.71)	36.2 (9.48)
BMI	18.3 (2.88)	18.1 (2.90)
Weight range (IOTF references) % (N)		
Obese	4.8 (23)	3.3 (15)
Overweight	15.9 (76)	17.2 (79)
Normal	76.6 (366)	74.9 (343)
Thin	2.7 (13)	4.6 (21)

Note – IOTF references were used, Cole et al., 2000 [33] and Cole et al., 2007 [34].

### Measurements

Information regarding students’ age, gender, school year and ethnic/cultural background (Table 1) was provided by means of a maternal telephone interview as well as a self report questionnaire completed by each student with closed response questions. Students were allocated an identification number and completed the questionnaire anonymously during regular class times each year under the supervision of the first two authors and trained research assistants.

Each mother participated in an annual telephone interview and provided details of the child’s date of birth, and parental details including parents’ age, current weight and height and highest level of father’s and mother’s education. Response categories included: ‘School certificate Year 10 or less’, ‘Higher School Certificate Year 12’, ‘Technical and Further Education College/Community College certificate’, ‘Undergraduate university or college degree’, and ‘Post graduate university degree’.

School SES information (Table 1) was obtained from the school principal and the department of education as low, middle or high. Individual student data for paternal and maternal educational level were collected (Table 1) as well as the national government school socioeconomic index [32]. The school SES category variable of low, middle or high school SES correlated with both the national government school socioeconomic index (Spearman rho = 0.82, p < 0.01) and the level of parental education (Spearman rho = 0.62, p < 0.01) and this variable was therefore selected as the measure of SES that was used in the analyses.

Height was measured to the nearest 0.5 centimetre without shoes using a portable free standing stadiometer (Harpenden PE038). Weight was measured to the nearest 0.1 kilogram using portable digital scales (Soehnle, Pharo 200). Students were measured annually during September – October in light school uniform, after removing shoes, jackets and emptying their pockets. BMI was calculated from measured heights and weights as weight (kg)/height (m)2 and obesity/overweight and thinness were defined using the International Obesity Task Force BMI cut-offs at 0.5 yearly intervals [33,34].

### Statistical methods

Data were analysed using SPSS version 21.0 [35]. The study sample provided the statistical power to calculated 95% confidence intervals (95% CI) in the range of 1.5% to 3% either side of prevalence estimates and to show that differences of 5-10% between groups eg SES groups were statistically significant (power = 80%, significance = <0.05). Continuous variables were summarised as the means and standard deviations (SD) and categorical variables were summarised as the percent (%) of the sample. Chi-square tests with a continuity correction were used to assess associations between categorical variables and trends over time. To undertake the longitudinal analysis to estimate whether the differences in SES groups over time were statistically significant, a linear mixed model was used. Different covariance structures were tested and an unstructured covariance matrix was found to provide the best fit. Least significant difference (LSD) tests were used to contrast the SES groups within time points. The residuals from the linear mixed model were plotted to verify that they were approximately normally distributed and to confirm that there were no influential outliers. Predicted mean values were plotted with 95% confidence intervals estimated as the standard error multiplied by 1.96. The study was approved each year by the University of Sydney Human Ethics Committee.

## Results

The demographic characteristics of the sample at baseline by gender are shown in Table 1. The total participants from Year 2 to Year 6 were well distributed across school years and gender. SES included 36% (n = 338) from low SES schools, 32.4% (n = 304) from middle SES schools, and 31.6% (n = 297) from high SES schools.

Weight status was categorised as obese, overweight, normal weight and thin in each study year by SES group and this is shown in Table 2. There was a significant difference in weight categories between SES groups in 2007 and 2011 with a higher percentage of children in the low SES groups who were obese or overweight. The pattern continued over 6 years. Chi-square trends tests showed no significant trends for either overweight or obesity to increase over the 6 years except for trend for obesity to increase in the mid SES group only (P < 0.01).

**Table 2:** Pattern of weight status in a longitudinal cohort of Australian school children of low, middle and high socioeconomic status between 2007 and 2012

**Year**	**2007**	**2008**	**2009**	**2010**	**2011**	**2012**
	**% (N)**	**% (N)**	**% (N)**	**% (N)**	**% (N)**	**% (N)**
Total N	935	814	758	738	772	695
Total sample						
Obese	4.1 (38)	3.6 (29)	4.4 (33)	4.5 (33)	4.8 (37)	6.0 (42)
Overweight	16.6 (155)	16.5 (134)	18.2 (138)	18.2 (138)	17.5 (135)	16.7 (116)
Normal	75.7 (708)	75.3 (613)	72.7 (551)	71.3 (526)	72.9 (563)	73.1 (508)
Thin	3.6 (3.4)	4.7 (38)	4.7 (36)	5.4 (40)	4.8 (37)	4.2 (29)
Low SES (N = 334)						
Obese	7.2 (24)	5.5 (16)	5.5 (15)	6.5 (16)	7.1 (19)	6.6 (15)
Overweight	15.8 (53)	16.7 (49)	18.9 (52)	20.2 (50)	19.1 (51)	21.0 (48)
Normal	73.4 (246)	72.4 (212)	72.7 (200)	70.2 (174)	71.5 (191)	68.6 (157)
Thin	3.6 (12)	5.5 (16)	2.9 (8)	3.2 (8)	2.2 (6)	3.9 (9)
Middle SES (N = 304)						
Obese	3.3 (10)	3.9 (10)	3.9 (9)	5.3 (12)	5.1 (12)	8.3 (18)
Overweight	17.1 (52)	17.4 (45)	20.4 (47)	19.4 (44)	16.9 (40)	16.1 (35)
Normal	75.0 (228)	74.0 (191)	69.1 (159)	69.2 (157)	71.2 (168)	71.4 (155)
Thin	4.6 (14)	4.7 (12)	6.5 (15)	6.2 (14)	6.8 (16)	4.1 (9)
High SES (N = 297)						
Obese	1.3 (4)	1.1 (3)	3.6 (9)	1.9 (5)	2.2 (6)	3.6 (9)
Overweight	16.8 (50)	15.2 (40)	15.4 (39)	17.1 (45)	16.4 (44)	13.3 (33)
Normal	79.1 (235)	79.8 (210)	75.9 (192)	74.1 (195)	75.8 (204)	78.7 (196)
Thin	2.7 (8)	3.8 (10)	5.1 (13)	6.8 (18)	5.6 (15)	4.4 (11)
Chi Square	16.3	9.7	7.3	11.0	13.8	10.5
P Value	0.01*	0.14	0.29	0.09	0.03*	0.10

*P<0.05.

Table 3 shows weight status in SES groups by gender. Results found that both males and females in low SES groups were more likely to be obese and overweight than middle and high SES groups. There was a significant difference between weight status and SES among females in 2008, with a higher percentage of low and middle SES groups who were obese and overweight (21.6% versus 20.9%) compared to high SES groups (16.2%). Overall, there was no significant gender difference in weight status over the 6 year study period.

**Table 3:** Change in weight status in a cohort of Australian school children from 2007 to 2012 by gender and SES

	**Males (N = 478)**							**Females (N = 458)**					
	**2007**	**2008**	**2009**	**2010**	**2011**	**2012**		**2007**	**2008**	**2009**	**2010**	**2011**	**2012**
	**% (N)**	**% (N)**	**% (N)**	**% (N)**	**% (N)**	**% (N)**		**% (N)**	**% (N)**	**% (N)**	**% (N)**	**% (N)**	**% (N)**
**Total sample**	478	403	383	367	395	361	**Total sample**	457	411	375	371	377	334
Obese	4.8 (23)	4.2 (17)	4.7 (18)	5.2 (19)	5.1 (20)	5.8 (21)		3.3 (15)	2.9 (12)	4.0 (15)	3.8 (14)	4.5 (17)	6.3 (21)
Overweight	15.9 (7.6)	16.1 (65)	17.2 (66)	17.4 (64)	16.2 (64)	17.2 (62)		17.3 (79)	16.8 (69)	19.2 (72)	20.2 (75)	18.8 (71)	16.2 (5.4)
Normal	76.6 (366)	75.2 (303)	73.6 (282)	73.3 (269)	74.7 (295)	73.7 (266)		74.8 (342)	75.4 (310)	71.7 (269)	69.3(257)	71.1 (268)	72.5(242)
Thin	2.7 (13)	4.5 (18)	4.4 (17)	4.1 (15)	4.1 (16)	3.3 (12)		4.6 (21)	4.9 (20)	5.1 (19)	6.7 (25)	5.6 (21)	5.1 (17)
**SES**							**SES**						
Low SES (N = 159)							Low SES (N = 175)						
Obese	8.2 (13)	5.1 (7)	5.3 (7)	8.0 (9)	7.1 (9)	4.6 (5)	Obese	6.3 (11)	5.7 (9)	5.6 (8)	5.2 (7)	7.1 (10)	8.3 (10)
Overweight	14.5 (23)	17.6 (24)	19.1 (25)	17.7 (20)	17.3 (22)	22.0 (24)	Overweight	17.0 (30)	15.9 (25)	18.8 (27)	22.2 (30)	20.7 (29)	20.0 (24)
Normal	74.8 (119)	74.3 (101)	73.3 (96)	71.7 (81)	74.0 (94)	69.7 (76)	Normal	72.2 (126)	70.7 (111)	72.2 (104)	68.9 (93)	69.3 (97)	67.5 (81)
Thin	2.5 (4)	2.9 (4)	2.3 (3)	2.7 (3)	1.6 (2)	3.7 (4)	Thin	4.5 (8)	7.6 (12)	3.5 (5)	3.7 (5)	2.9 (4)	4.2 (5)
Middle SES (N = 161)							Middle SES (N = 143)						
Obese	4.3 (7)	5.2 (7)	5.1 (6)	5.9 (7)	5.7 (7)	9.5 (11)	Obese	2.1 (3)	2.4 (3)	2.7 (3)	4.6 (5)	4.4 (5)	6.9 (7)
Overweight	17.4 (28)	16.4 (22)	18.8 (22)	19.3 (23)	17.9 (22)	17.2 (20)	Overweight	16.8 (24)	18.5 (23)	22.1 (25)	19.4 (21)	15.9 (18)	14.9 (15)
Normal	75.8 (122)	73.9 (99)	70.9 (83)	72.3 (86)	71.5 (88)	71.6 (83)	Normal	74.1 (106)	74.2 (92)	67.3 (76)	65.7 (71)	70.8 (80)	71.3 (72)
Thin	2.5 (4)	4.5 (6)	5.1 (6)	2.5 (3)	4.9 (6)	1.7 (2)	Thin	7.0 (10)	4.8 (6)	8.0 (9)	10.2 (11)	8.8 (10)	6.9 (7)
High SES (N = 158)							High SES (N = 139)						
Obese	1.9 (3)	2.3 (3)	3.7 (5)	2.2 (3)	2.8 (4)	3.7 (5)	Obese	0.7 (1)	0 (0)	3.4 (4)	1.6 (2)	1.6 (2)	3.5 (4)
Overweight	15.8 (25)	14.3 (19)	14.1 (19)	15.6 (21)	13.8 (20)	13.2 (18)	Overweight	18.0 (25)	16.2 (21)	16.9 (20)	18.8 (24)	19.4 (24)	13.3 (15)
Normal	79.1 (125)	77.4 (103)	76.3 (103)	75.6 (102)	77.9 (113)	78.7 (107)	Normal	79.1 (110)	82.3 (107)	75.4 (89)	72.7 (93)	73.4 (91)	78.8 (89)
Thin	3.2 (5)	6.0 (8)	5.9 (8)	6.7 (9)	5.5 (8)	4.4 (6)	Thin	2.2 (3)	1.5 (2)	4.2 (5)	7.0 (9)	5.6 (7)	4.4 (5)
Chi Square	7.4	3.9	4.1	8.2	6.8	8.9		12.4	15.2	5.6	7.2	9.4	6.0
P value	0.29	0.70	0.67	0.22	0.34	0.18		0.054	0.02*	0.47	0.30	0.15	0.42

*P<0.05.

**Figure 1:**
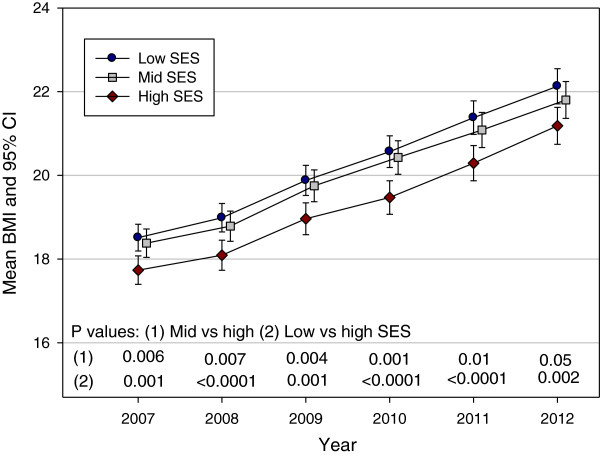
**Pattern of mean Body Mass Index (BMI) in a cohort of Low, Middle and High SES Australian school children from 2007 to 2012.** Note - 95% confidence intervals and P values compare Middle SES versus High SES (1) children and Low SES versus High SES children (2).

**Figure 2:**
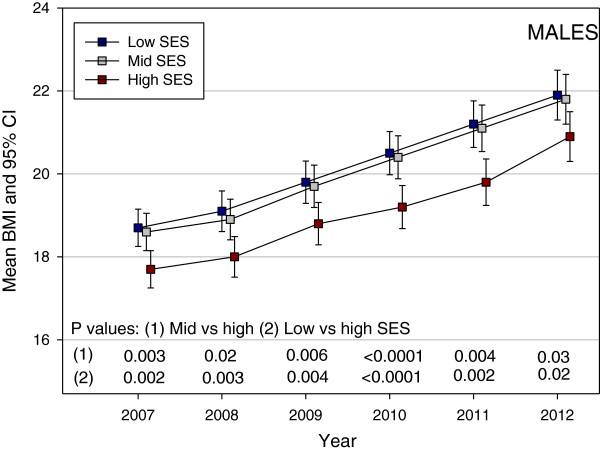
Pattern of mean Body Mass Index (BMI) in a cohort of Low, Middle and High SES male Australian school children from 2007 to 2012.

**Figure 3:**
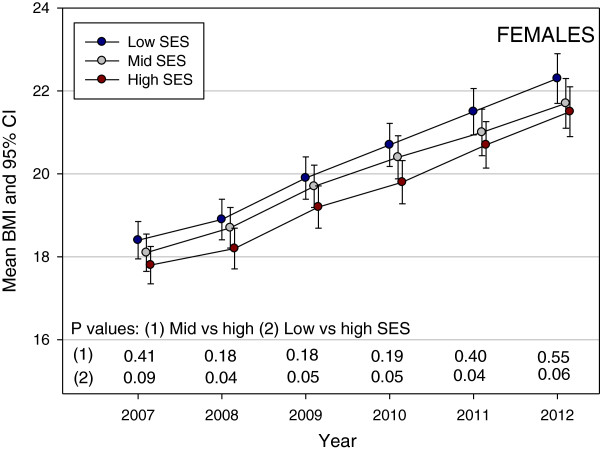
Pattern of mean Body Mass Index (BMI) in a cohort of Low, Middle and High SES female Australian school children from 2007 to 2012.

Figure 1 shows the predicted mean BMI by year and SES from the linear mixed model. Gender was not significant in the model; the mean difference between genders was 0.24 kg/m^2^ (95% CI âˆ’0.13, 0.61); P = 0.21. However, both Year of the study (P < 0.0001) and SES (P = 0.003) were significant predictors of mean BMI. An interaction between Year and SES was tested but was not significant (P = 0.29) and did not improve the fit of model. The P values for the contrasts between mid and high SES and between low and high SES are shown in Figure 1. All of the contrasts were significant with the high SES group having a consistently lower BMI than the mid and low SES groups. The difference between low and high SES remained constant and the mean BMI in mid SES became closer to the mean BMI in high SES over time. When the models were examined for males and females separately (Figures 2 and 3), the patterns of change in BMI over time appeared slightly different. In males (Figure 2), the mean values for low and mid SES remained close and were significantly higher than for high SES with no convergence of groups over time. For females (Figure 3), the lines for the three SES groups were separated and the mid SES group converged with the high SES group over time. However, post hoc tests showed no statistically significant gender differences between SES groups at any time point.

## Discussion

The purpose of this large longitudinal study was to examine the prevalence of thinness, overweight and obesity in a longitudinal cohort of schoolchildren from three large school regions of regional NSW, from 2007–2012, in order to examine how the socioeconomic gradient in weight status, namely thinness, overweight and obesity, changes over time. We found a consistent association between SES and overweight and obesity prevalence over the 6-year period, with a higher percentage of children from low SES categorized as overweight or obese, compared to other SES groups. This suggests that socio-economic factors are likely to determine obesity and overweight in children and that these social determinants of health are well established by primary school age (as early as 8 years of age) and continue to be influencing factors into adolescence. Further, the greater prevalence in obesity among children from low SES schools was consistent across both genders. The high SES female group had a consistently lower BMI than the mid and low SES female groups. The difference between low and high SES females remained constant and the mean BMI in mid SES became closer to the mean BMI in high SES over time. In males, the mean values for low and mid SES remained close and were significantly higher than high SES males, with no convergence of groups over time. This suggests a potential future risk of overweight and obesity in adulthood and related ill-health concerns for children and adolescents from socially disadvantaged, low income communities and schools.

This low SES trend has also been reported in similar cross sectional studies in Australia [21,28-30,36,37], as well as international studies from developed countries [24,38-41]. There have been few longitudinal studies of obesity trends in children but those that have been reported have found similar trends by SES [17,20,42].

A recent paper by Griffiths et al., [43] sheds light on the importance of measuring waist circumference as well as body mass index, as their study found a plateau in BMI, but an increase in waist circumference, especially in girls, and that central adiposity, measured by weight circumference is increasing alongside a stabilization in BMI. It is possible therefore, that as in the recent English cohort study, Australian and other Westernised children may be getting fatter and the additional adiposity may be stored centrally which is not detected by BMI. This methodological consideration is important and therefore warrants further research using various measures of weight status as well as adiposity.

The impact of ethnicity on the prevalence of obesity has been reported in several larger studies [44-49] and supports the current study’s findings in regard to a higher prevalence of overweight and obesity among Pacific Islander youth. It has been suggested that muscularity and height may be reflected in BMI and obesity prevalence of Pacific Islander adults [47-49] and children [46,50,51]. However, a more recent study reports that a higher rate of obesity and its sequelae seen in Pacific Islanders in Hawaii may be more as a result of socioeconomic status and lifestyle than of genetic propensity [45]. These ethnic and/or cultural trends warrant further investigation with greater numbers of participants from various ethnic groups to tease out the individual impacts of genetics, social determinants of weight and cultural mores.

An interesting component of the current study is the inclusion of thinness as an indicator of weight status over time. Only one other Australian study has included a thinness outcome [28], and the authors reported a cross-sectional finding of stability in the prevalence of thinness over time. This is an important finding, as there has been a level of concern that the increasing number of childhood and adolescent overweight and obesity intervention programs over the previous 10 years may have caused unintended psychological and physiological harm, promoted internalization of the thin-ideal, and heightened the risk of body image concerns and eating disorders [52].

The strengths of the current study include a large representative longitudinal cohort from New South Wales (Australia) with a very high baseline participation rate; high retention rates over the 6 year period; measured height and weight every year; and strong independent SES indicators of parental education, the government school index and school SES status. In light of these strengths, there a small number of study limitations that should be acknowledged. First, there were small numbers of participants from various ethnic backgrounds, such as Aboriginal and Pacific Islander children. This meant that the impact of ethnicity or culture on obesity, overweight or thinness prevalence could not be confirmed in this longitudinal sample because of low cell numbers in analyses. Second, a potential limitation was selection bias which arises from non-participation [53]. We can report that non-participation was mainly in regard to administration issues (e.g. parents not receiving consent forms) as well as some parents declining consent because their child was intellectually disabled, or had a medical condition such as Type 1 diabetes, coeliac disease, an eating disorder or other illness. These parents reported, via telephone interview, that they decided not to expose their child to any stigma or stress in regard to completing the survey questions. A total of 40 students participated in 2007, but did not participate after this, with these students moving interstate or overseas and were unable to be contacted. Data on those who did not consent to participate, or who withdrew from the study during the 6 year period did not differ to other participants who continued to participate in the longitudinal study. Another limitation is the lack of a measure of central adiposity, such as waist circumference.

The reasons behind the clear socioeconomic trends need to be further examined to assess what is protective in high SES communities and whether measurement using BMI is largely reflective of ethnic body composition or not. Targeted interventions appear to be necessary on a social disadvantage platform and should be developed in collaboration with community members and youth in order to avoid unintended stigma, criticism of cultural body ideals and norms and other potentially harmful outcomes.

## Conclusions

Patterns of obesity and overweight in children from socially and economically disadvantaged communities in regional NSW are identifiable from a young age and the socioeconomic pattern persists into adolescence. Obesity prevention and intervention programs should be designed, implemented and evaluated with the social determinants of health in mind and in collaboration with community members. Community programs should continue to be based on positive rather than negative messages in order to avoid unintended stigma and other potentially harmful outcomes.

## Competing interests

The authors declare that they have no competing interests.

## Authors’ contributions

JOD designed and carried out the longitudinal study, performed the statistical analyses and drafted the manuscript. HC conducted research data collection, performed data entry assisted in the literature review and assisted in drafting the manuscript. LP assisted with the literature review, assisted in drafting the manuscript and wrote the Introduction. All authors contributed to and approved the final manuscript.

## Pre-publication history

The pre-publication history for this paper can be accessed here:

http://www.biomedcentral.com/1471-2458/14/222/prepub
